# Management of traumatic wounds in the Emergency Department: position paper from the Academy of Emergency Medicine and Care (AcEMC) and the World Society of Emergency Surgery (WSES)

**DOI:** 10.1186/s13017-016-0084-3

**Published:** 2016-06-18

**Authors:** Carolina Prevaldi, Ciro Paolillo, Carlo Locatelli, Giorgio Ricci, Fausto Catena, Luca Ansaloni, Gianfranco Cervellin

**Affiliations:** Emergency Department, Hospital of San Donà di Piave VE, Parma, Italy; Emergency Department, Academic Hospital of Udine, Parma, Italy; Institute of Toxicology, IRCCS Fondazione Maugeri Pavia, Parma, Italy; Emergency Deparment, Academic Hospital of Verona, Parma, Italy; Emergency Surgery, Academic Hospital of Parma, Parma, Italy; Emergency surgery, Hospital of Bergamo, Parma, Italy; Emergency Department, Academic Hospital of Parma, Parma, Italy

**Keywords:** Traumatic wounds, Infection, Foreign body, Tetanus, Rabies, Suture

## Abstract

Traumatic wounds are one of the most common problems leading people to the Emergency Department (ED), accounting for approximately 5,4 % of all the visits, and up to 24 % of all the medical lawsuits. In order to provide a standardized method for wound management in the ED, we have organized a workshop, involving several Italian and European experts. Later, all the discussed statements have been submitted for external validation to a multidisciplinary expert team, based on the so called Delphi method. Eight main statements have been established, each of them comprising different issues, covering the fields of wound classification, infectious risk stratification, tetanus and rabies prophylaxis, wound cleansing, pain management, and suture. Here we present the results of this work, shared by the Academy of Emergency Medicine and Care (AcEMC), and the World Society of Emergency Surgery (WSES).

## Introduction

Traumatic wounds are one of the most common problems leading people to the Emergency Department (ED), and account for approximately 5,4 % of all the visits [1, 2]. The ED represents the most available facility for wound care, due to the 24-h free access and the decreasing primary care availability. As such, provision for effective and safe wound care will continue to be a priority for Emergency Physicians (EPs). Moreover, traumatic wounds have been historically a major source of litigation against EPs, accounting for up to 24 % of all the medical lawsuits, mainly due to missed identification and treatment of tendon or nerve injuries, or to infection and/or presence of foreign bodies [2]. Hence, although most wounds will heal without any treatment, a prompt and careful repair of these injuries reduces infection and scarring, so improving the patient satisfaction and avoiding significant additional costs [1]. However, in current clinical practice several different approaches to traumatic wounds are still practiced, due to cultural gaps, myths and local traditions.

One of the specific goals of the third European Union (EU) program in the health care area, years 2014–2020, is to improve access to a skilled, standardized and safe health care for EU citizens, thus improving the quality of health care and patient safety. According to these objectives we have organized a workshop aimed to share knowledge and experiences in the field of wound care, involving several Italian and European experts. The workshop was settled in Venice, in October 2014. Later, all the discussed statements have been submitted for external validation to a multidisciplinary expert team, as described in the methods. On the basis of the results of this complex and time-consuming work, the Academy of Emergency Medicine and Care (AcEMC), and the World Society of Emergency Surgery (WSES) have decided to build, write and spread a multidisciplinary position statement on the management of traumatic wounds in the ED.

The main purposes of the present work are:

- To assess the current scientific evidence on the subject.
- To draw up a multidisciplinary consensus document aimed to establish a standardized and correct method of management of traumatic wounds in the ED.
- To help clinicians in the clinical risk stratification, to improve diagnostic and therapeutic appropriateness as well as the cost-benefit ratio, to reduce clinical errors, and to increase patient satisfaction.
- To provide an opportunity for research and educational initiative.

## Methods

We have decided to use a modified Delphi method, that is a structured communication technique, originally developed as a systematic, interactive forecasting method which relies on a panel of experts [3, 4]. The experts answer to one ore more questionnaires in two or more rounds. After each round, a facilitator provides an anonymous summary of the experts’ forecasts from the previous round as well as the reasons they provided for their judgments. Thus, experts are encouraged to revise their earlier answers in light of the replies of other members of the panel. It is expected that during this process the range of differences of the answers will decrease and the group will converge towards the “correct” answer. Finally, the process is stopped after a pre-defined stop criterion (e.g. number of rounds, achievement of consensus, stability of results) and the mean or median scores of the final rounds determine the results [5].

The Delphi method is based on the principle that forecasts or decisions obtained from a structured group of individuals are more accurate than those from unstructured groups [6]. The name “Delphi” derives from the Oracle of Delphi, thus carrying in itself a somewhat mythical nuance. However, the method was developed at the beginning of the Cold War to forecast the impact of technology on warfare [6]. One of the key characteristics of the method relies on the anonymity of the participants. As such, usually all participants remain anonymous, at least until the completion of the final report. This prevents the authority, personality, or reputation of some participants from dominating others in the process. Another important key characteristic is the regular feedback given to the participants, so that they can know comments on their own forecasts, as well as the responses of others, and the progress of the panel as a whole. The last key characteristic relies on the role of the facilitator, i.e. the person coordinating the group. He/she facilitates the responses of their panel of experts, collects and analyzes them, thus identifying the conflicting viewpoints. If consensus is not reached, the process continues through thesis and antithesis, to gradually work towards synthesis, and building consensus.

To build this document we have composed a multidisciplinary panel consisting of EPs and Surgeons, as well as other experts in different fields, coming from different countries. The study, which lasted about four months, was divided into two different phases. In both phases a dedicated questionnaire was sent by e-mail to each member of the panel. In the first phase, there were three rounds. After that, consensus was reached in eight of the topics addressed. as such, it was considered as appropriate, in the second step, to repeat the round in order to try to reach consensus on all the addressed issues. The external validation of the document was reached organizing a two days’ workshop, inviting a group of European experts to discuss and validate the statements [7, 8]. See Appendix.

As such, the first step was based on a series of key questions, as reported in Table 1.

**Table 1:** The questions submitted to the experts

Can you define “clean” a traumatic wound in the setting of the emergency department?
What is your approach to the prophylaxis of wounds with a high risk of infection (e.g., bites, wounds of the hand/foot…)?
Do exist, and, if yes, how reliable are the signs predictive of risk of infection?
Your opinion on methods of prevention of infection: irrigation, closure technique, antibiotic prophylaxis.
In such wounds do you consider as appropriate to assess the status of immunization against tetanus?
Do you consider appropriate the classification of traumatic wounds “clean wound not tetanigenic”?
Since it has been shown that only about 15 % of patients with traumatic wounds carry with them the documentation on their own tetanus immunization status, as noted by the vaccination status of patients prior to tetanus prophylaxis?
Have you ever had difficulties during the anamnesis to assess the state of tetanus vaccine injured patients who present to the emergency department?
Since only 15 % of patients present with documented data on vaccinations and health registry is rarely accessible from the emergency room, in the absence of data, trusts the patient’s history on their vaccination status?
If you had to provide a quick diagnostic test to evaluate immediately and with certainty immunization status of injured patients compared to tetanus, would consider it useful in the emergency department to improve the appropriateness of tetanus immunoprophylaxis and management of his patients?

## Results

### Definitions

At the end of the work the panel and the referees have reached an agreement on the following definitions of traumatic wounds:

- **Traumatic Wound**: a wound or laceration of traumatic origin with no evidence of macroscopic contamination or signs of active infection (and likely low probability of infection).
- **Dirty Traumatic Wound**: a wound or laceration of traumatic origin macroscopically contaminated. Among these wounds we include those with simultaneous perforation of a viscus; with presence of devitalized tissues; with foreign bodies; those that occurred in a contaminated environment (dung, marshes); animal bites; puncture wounds; wounds with a delayed treatment.
- **Infected Traumatic Wound**: a wound or laceration of traumatic origin with signs of infection (secretions) [9–13].

After completed that step, the panel reached consensus on a series of statements concerning the management of traumatic wounds. For each statement, selected references are provided. The statements are as follows:

- **STATEMENT 1**
  All traumatic wounds are to be considered contaminated at presentation in ED.

- **STATEMENT 2**
  It is useful to provide an initial stratification of the risk of infection for all the traumatic wounds. The risk assessment should be based on both the following: i) type of wound; ii) location of the wound; iii) characteristics of the wounded patient.
  With the aim of simplifying and optimizing the management of patients in the ED, the following fields of stratification of the risk of infection was identified: type of wound, location of the wound, characteristics of the patients. In Tables 2, 3, 4 the suggested items for risk assessment are summarized.

**Table 2:** Infection risk assessment based on type of wound

Straight stab wounds	low risk
Tears/bruises/contusion wounds	high risk
Puncture wounds	high risk
Wound with crush injuries	high risk
Bite wounds	high risk
Wounds contaminated with feces	high risk
Wounds contaminated with soil and dirt, or mineral oil	high risk
Wounds with the presence of foreign bodies	high risk
Wounds with edge diastasis	high risk
Engagement of deep tissues, exposed fracture	high risk

**Table 3:** Infection risk assessment based on the location of the wound

Well vascularized tissue (head, neck, scalp)	low risk
High concentration of commensal flora (oral mucosa, genitals, armpits)	high risk
Poorly vascularised (hand, foot, lower and upper limb)	high risk

**Table 4:** Infection risk assessment based on the characteristics of the patient

Child	low risk
Young	low risk
Adult	low risk
Elderly (>65 years)	high risk
Immunocompromised (treated with steroids, immunosuppressive agents, splenectomised, HIV …)	high risk
Vascular disease	high risk
Diabetic	high risk

  - **2A.** Avoid antibiotic administration in low risk wounds (for all three variables considered).
  - **2B.** Consider antibiotic administration when one or two high risk variables are present.
  - **2C.** If the decision to avoid antibiotic administration in high risk wounds is made the reason must always be clearly stated.
  - **2D.** In every wound consider the risk of tetanus according to the patient’s immunization status.

- **STATEMENT 3**
  **Antibiotic prophylaxis** (i.e., a preventive administration of an antibiotic before the emergence of an infection with the aim to prevent it). It is desirable to implement prophylactic antibiotics in selected cases of wounds at high risk of infection.
  - **3A.** Avoid antibiotic prophylaxis in a not macroscopically contaminated wound, well vascularized, at low risk of infection (according to statements 2).
  - **3B.** Antibiotic prophylaxis should be considered in grossly contaminated wounds and in cases at high risk of infection (according to statement 2) depending on the epidemiological criteria of antibiotic resistance in the area. In high risk wounds (all three variables considered) the EP should explain clearly the reason for avoiding the antibiotic administration [14–19].

- **STATEMENT 4**
  The assessment of tetanus immunization status in every traumatic wounded patient who arrive in the ED is desirable.
  - **5A.** All traumatic wounds are potentially at risk for tetanus infection
  - **5B.** The assessment of tetanus immunization status of patients should be performed through a thorough history and consultation of documentation confirming vaccination/booster, and eventually using a diagnostic quick test in doubtful cases.
  - **5C.** The following items should be considered as “doubtful” (i.e., cases for which it is not possible to determine the immunization status of the patient):
    - Patient who does not remember the date of the last booster;
    - Patient unconscious, intoxicated or cognitively impaired;
    - Patient who does not understand your language;
    - Patient who, presumably, has never carried out a complete vaccination course.
  - **5D.** Access to vaccination data and the availability of a rapid diagnostic test for assessing the status of tetanus immunization permit to streamline costs and to act with greater appropriateness [20–23].

- **STATEMENT 5**
  It is desirable that in any ED is available the first administration of rabies vaccine (for at least two patients).
  Doses sufficient for full courses of rabies immunoglobulin treatment for two patients should be available in Poison Control Centers and in 2nd level EDs (at least 1 for every 5 million inhabitants and at least 1 in each major island) [24, 25].

- **STATEMENT 6**
  It is desirable a proper and timely implementation of procedures and methods for preventing infection in any traumatic wound. The identified methods of preventing infection are the following:
  - **6A.** Irrigation using appropriate security safeguards. Irrigation can be performed with saline (or tap water), with high pressure if necessary, according to the degree of contamination of the wound and the anatomic location.
  - **6B.** Search for foreign bodies. Beside an accurate visual inspection, X-rays, CT or ultrasound examination should be taken into consideration.
  - **6C. Suture technique**
    - ✓ Avoid shaving of hair
    - ✓ With simple stitches, always after irrigation
    - ✓ The intradermal suture should be avoided in most cases
    - ✓ If the risk of infection is high suture may be delayed [26–28].

- **STATEMENT 7**
  All the wounds of the hand should be carefully evaluated, considering them at high risk of error.
  - **7A**. Any traumatic injury of the hand should be considered for a possible tendon injury, especially if located on the volar or dorsal side.
  - **7B.** Any traumatic injury of the hand should be considered for a nerve injury, especially if located on the lateral side of the fingers
  - **7C.** A physical examination should be performed in any traumatic injury of the hand to check for any eventual tendon or nerve damage before performing the anaesthesia.
  - **7D.** In every traumatic injury of the hand treated in the emergency department the possibility of performing a follow-up should be considered [29–38].

- **STATEMENT 8**
  It is a priority to treat pain in traumatic wounds in all patients who attend to the ED. Several different protocols for the pain management are available, both pharmacological and non-pharmacological. Oral, local, intravenous, intra-nasal, and respiratory way (i.e., nitrous oxide) may be taken into consideration [39].

## Conclusions

We consider our work as a starting point and networking opportunity for participation in the forthcoming call funding programs in health care. In addition, the shared document (position paper), validated during the workshop with the precious contribution of international experts, intends to contribute to policy and health priorities in the European and international areas.

## Appendix

Writing committee members: Francesca Venturi Visconti, Rome; Massimo Pesenti Campagnoni, Aosta; Ivo Casagranda, Alessandria; Pierdante Piccioni, Codogno; Daniele Coen, Milan; Massimo Crapis, Udine; Andrea Rocchetti, Alessandria; Egidio Barbi, Trieste; Augusto Tricerri, Rome; Libero Barozzi, Bologna; Fabio Brunato, Padova; Mario Cavazza, Bologna; Marco Ricca, Cuneo; Massimo Rega, Cuneo; Pasquale Picciano, Jesolo; Stefano M. Calderale, Rome; Alberto Albani, Rome; Donatella Del Gaizo, Neaples; Andrea Drei, Faenza; Fabrizio Giostra, fermo; Franco Laterza, San Donà di Piave; Luigi Zulli, Rome; Maria Giuliano, Neaples; Francesca Velluti, San Donà di Piave; Carlo Manfredi, Firenze; Maria Carolina Barbazza, San Donà di Piave.

External referees: Nikolaos K Paschos, Joannina, Greece; Miguel Angel Bratos, Valladolid, Spain; Federica Norat, Nice, France; Fabio Toffoletto, San Donà di Piave, Italy; Rodolfo Sbrojavacca, Udine, Italy; Bruno Mégarbane, Paris, France; Biagio Epifani, Mirano, Italy; Camilla Negri, Gorizia, Italy; Matteo Pistorello, Montebelluna, Italy; Michael Espa, Lyon, France; Cavenaile Jean-Christophe, Bruxelles, Belgium; Primo Botti, Firenze, Italy; Paola De Benedictis, Legnaro, Italy; Roberta Aiello, Legnaro, Italy; Marta Mazzoleni, Pavia, Italy; Michele Alzetta, Venezia, Italy; Michele Mitaritonno, Parma, Italy; Arianna Fede Catania, San Donà di. Piave, Italy; Antonella Tonetto, San Donà di. Piave, Italy; Farhadullah Khan, San Donà di. Piave, Italy; Buffolo Gabriella, San Donà di. Piave, Italy; Flavia Gandin, Udine, Italy; Maria Rita Laera, Alessandria, Italy; Cesare Montecucco, Padova, Italy; Lorenzo Calligaris, Trieste, Italy; Peter Heinz, Cambridge, UK; Tiziana Zangardi, Padova, Italy; Maria Paola Saggese, Brescia, Italy; Mario Saia, Venice, Italy; Fabio De Jaco, Imperia, Italy; Francesco Pratticò, Verona, Italy; Roberto Lerza, Savona, Italy; Guido Grazie, Savona, Italy; Liviana Da Dalt, Padova, Italy; Almerto De Mas, Pordenone, Italy.
